# Method Comparison of PT, INR, and aPTT Results Obtained Using Two Coagulation Analyzers in Routine Laboratory Practice

**DOI:** 10.3390/diagnostics16182989

**Published:** 2026-09-15

**Authors:** Betül Özbek Kurtul, Bağnu Dündar, Gül İpek Gündoğan, Sevgi Kocyigit Sevinc, Tuğba Elgün, Asiye Gök Yurttaş

**Affiliations:** 1 Department of Medical Biochemistry, Faculty of Medicine, Istanbul Atlas University, 34408 Istanbul, Türkiye; betul.ozbek@atlas.edu.tr (B.Ö.K.); bagnu.dundar@atlas.edu.tr (B.D.); 2 Department of Medical Biology, Faculty of Medicine, Biruni University, 34015 Istanbul, Türkiye; ggundogan@biruni.edu.tr (G.İ.G.); telgun@biruni.edu.tr (T.E.); 3 Biruni University Research Center (BAMER), Biruni University, 34015 Istanbul, Türkiye; 4 Department of Biophysics, Faculty of Medicine, Kütahya Health Sciences University, 43020 Kütahya, Türkiye; sevgi.kocyigitsevinc@ksbu.edu.tr

**Keywords:** prothrombin time, international normalized ratio, activated partial thromboplastin time, coagulation analyzer, method comparison, Bland–Altman, Passing–Bablok, analytical bias

## Abstract

**Background/Objectives:** Prothrombin time (PT), international normalized ratio (INR), and activated partial thromboplastin time (aPTT) are routinely used coagulation assays, and analyzer–reagent differences may affect result comparability. This study evaluated analytical comparability between the Sysmex CS-2500 System using Siemens reagents and the Tokra Medical NOVAE II for PT, INR, and aPTT. **Methods:** Residual routine citrated plasma specimens were measured on both systems. The CS-2500 was designated as the comparator system. Passing–Bablok regression and Bland–Altman analysis were used as the primary method-comparison approaches; Pearson and Spearman correlations and exploratory categorical agreement were secondary analyses. **Results:** Fifty paired measurements were analyzed for PT and INR and 54 for aPTT. For all three parameters, the 95% confidence interval (CI) for the Passing–Bablok intercept included 0 and the 95% CI for the slope included 1, providing no statistically supported evidence of constant or proportional bias by regression. Mean paired differences (NOVAE II minus CS-2500) were +1.27 s for PT, +0.023 for INR, and +2.32 s for aPTT. The 95% limits of agreement were −0.15 to +2.69 s for PT, −0.112 to +0.159 for INR, and −1.70 to +6.33 s for aPTT. Categorical agreement was influenced by analyzer-specific reference intervals and by the low prevalence of abnormal results. **Conclusions:** The two analyzer–reagent systems showed positive analytical associations, but the magnitude and dispersion of paired differences varied by assay. Because no clinical equivalence margins were prespecified and markedly pathological or therapeutic-range samples were sparsely represented, these findings support analytical comparison and local verification but do not establish clinical interchangeability.

## 1. Introduction

Prothrombin time (PT), international normalized ratio (INR), and activated partial thromboplastin time (aPTT) remain among the most frequently requested assays in clinical hemostasis laboratories, serving three principal clinical purposes: monitoring of anticoagulant therapy (vitamin K antagonists for PT/INR and unfractionated heparin for aPTT), initial evaluation of unexplained bleeding, and, despite ongoing debate regarding diagnostic yield, routine preoperative screening [1]. Although several major clinical guidelines have questioned the value of coagulation screening as a blanket preoperative tool—arguing that a carefully obtained bleeding history and physical examination are often more informative than isolated PT/aPTT results in patients without a known bleeding tendency—these assays continue to be ordered at high volume in everyday practice, underscoring their entrenched role in perioperative and hemostatic risk assessment [2]. Beyond preoperative contexts, PT/INR and aPTT retain clear clinical utility in monitoring therapeutic anticoagulation, evaluating hepatic synthetic function, and investigating suspected congenital or acquired coagulation factor deficiencies, which reinforces the need for these assays to be both accurate and reproducible across the analytical platforms on which they are performed. Although PT, INR, and aPTT assays are now highly automated and widely available across clinical laboratories, their numerical outputs are not universally interchangeable between instruments. Result variability can arise from several interrelated sources, including the specific instrument–reagent combination employed, the underlying clot detection technology (mechanical/electromechanical versus optical/photo-optical methods), calibration procedures and the international sensitivity index (ISI) assigned to the thromboplastin reagent, preanalytical handling conditions such as sample collection, citrate ratio, and time-to-analysis, and analyzer-specific reference intervals [3,4,5]. These sources of variability are not merely theoretical: several recent comparative evaluations of automated coagulation analyzers have demonstrated that, even when individual devices meet acceptable internal analytical performance criteria in terms of precision and linearity, clinically relevant method-related differences can still emerge when the same routine hemostasis parameters—most notably PT and aPTT—are measured in parallel on different platforms [6,7,8,9].

A substantial body of literature has examined whether clot-detection technology contributes to inter-instrument discordance. Comparative studies of mechanical, electromechanical, optical, and photo-optical coagulometers generally report positive correlations for PT, INR, and aPTT while also identifying method-related differences and finite limits of agreement, particularly for aPTT [10]. These observations emphasize that correlation alone describes association and does not demonstrate agreement or clinical interchangeability. Regression-based approaches such as Passing–Bablok regression and Bland–Altman analysis provide complementary information on constant or proportional differences, average paired differences, and the dispersion of those differences [11,12]. Current haemostasis guidance therefore emphasizes evaluation of the complete analyzer–reagent test system and recommends local comparability studies using patient samples that span the clinically relevant reportable range [3].

Published independent data directly comparing the CS-2500 and Tokra NOVAE II analyzer–reagent systems are limited. The present study was therefore designed as a paired patient-sample method-comparison study to quantify analytical comparability and the direction and magnitude of paired differences for PT, INR, and aPTT under routine laboratory conditions. The CS-2500 was used as the comparator system, and NOVAE II values were evaluated against it using Passing–Bablok regression, Bland–Altman analysis, association statistics, and exploratory normal/abnormal classification. The study was not designed to establish a new reference measurement procedure or to demonstrate clinical interchangeability.

## 2. Materials and Methods

### 2.1. Study Design and Sample Selection

This prospective paired method-comparison study evaluated PT, INR, and aPTT results obtained with the Sysmex CS-2500 (Sysmex Corporation, Kobe, Japan) and Tokra Medical NOVAE II (Tokra Medical, Ankara, Türkiye) coagulation analyzers. Residual citrated plasma specimens were prospectively selected after completion of the requested routine laboratory analyses; no additional venipuncture was performed specifically for the method-comparison study. Sample selection was performed irrespective of patient diagnosis. Samples showing visible hemolysis, lipemia, icterus, clots, or underfilling or overfilling of the citrate tube were excluded. Each eligible specimen was assigned a unique study code, and only age and sex were retained; clinical diagnosis, coagulation-disorder status, and anticoagulant treatment were not retained for subgroup analysis.

Venous blood samples were collected into 1.8-mL VACUSERA^®^ sodium citrate coagulation tubes (Disera Tıbbi Malzeme Lojistik San. ve Tic. A.Ş., İzmir, Türkiye) containing 3.2% buffered sodium citrate (0.109 mol/L), with a blood-to-citrate ratio of 9:1. Samples were transported to the central laboratory either via the pneumatic tube system or manually by clinical staff. Following routine specimen-acceptance procedures, samples were centrifuged at 1500× *g* for 15 min at room temperature. Samples remained in their original primary citrate tubes at a laboratory temperature of 19–22 °C throughout routine testing and until the corresponding results had been reviewed and authorized. Collection times were subsequently verified through the laboratory information system (LIS), and only specimens for which routine testing and result authorization had been completed within 2 h of collection were eligible for the study.

Study measurements were performed directly from the same primary tube without aliquoting or transfer to a secondary tube. Paired measurements on the CS-2500 and NOVAE II were performed sequentially; immediately after sample aspiration was completed and the primary tube was released by the first analyzer, it was transferred directly to the second analyzer. No fixed analyzer was designated to be tested first, and the testing order varied according to routine workflow. Results obtained from both analyzers were recorded in the study data sheet.

The study was conducted in accordance with the Declaration of Helsinki and was approved by the Istanbul Atlas University Non-Interventional Clinical Research Ethics Committee (meeting no. 06, decision no. 06, approval no. E-22686390-200-106841, 22 June 2026).

### 2.2. Analyzer–Reagent Systems, Calibration, and Quality Control

The CS-2500 was designated as the comparator system because it was the laboratory’s established routine coagulation platform against which the NOVAE II system was evaluated. This designation does not imply metrological reference status. For noncalibrated coagulation screening tests such as PT and aPTT, a true-value standard is not available; current guidance therefore supports assessment of comparability against an established or predicate analyzer–reagent test system [3].

The CS-2500 uses a multiwavelength photo-optical clot-detection principle. PT was measured using Siemens Dade^®^ Thromborel^®^ S (lot 576544; Siemens Healthcare Diagnostics Products GmbH, Marburg, Germany), a lyophilized human placental thromboplastin reagent. The lot-specific ISI used for the Thromborel^®^ S–CS-2500 combination was 1.05. aPTT was measured using Siemens Dade^®^ Actin^®^ FS Activated PTT Reagent (lot 562506; Siemens Healthineers), which uses ellagic acid as the activator and soybean phosphatides as the phospholipid source. INR was calculated using the standard INR relationship based on the reagent/system-specific ISI and mean normal prothrombin time (MNPT). The laboratory-specific MNPT was determined using PT measurements from 20 normal reference individuals and was 11.58 s.

The NOVAE II uses an optical clot-detection principle. PT was measured using MTI PT ThromboPlast (lot 01434801; Tokra Medical, Ankara, Türkiye), a liquid rabbit-brain-derived thromboplastin reagent containing calcium, with a lot-specific ISI of 1.15. aPTT was measured using MTI-1120 APTT S (lot 01804102; Tokra Medical), a liquid reagent containing a phospholipid suspension and a particulate contact activator composed of magnesium, aluminum, and silicate; calcium chloride was used to initiate the clotting reaction. INR was calculated according to the manufacturer’s recommended relationship using patient PT and the reagent/system-specific ISI. The analyzers were evaluated using their respective manufacturer-specific reagent systems; therefore, the comparison represents the performance of each analyzer–reagent system as implemented during the study. PT and aPTT results were reported in seconds, whereas INR was reported as a unitless calculated value.

Internal quality control was performed for both systems using manufacturer-recommended normal and pathological control materials, and study measurements were initiated only after both control levels met the predefined manufacturer acceptance criteria. The study was conducted over a 4-day period. On each study day, normal and pathological control levels were analyzed on both systems before patient-sample testing. In addition, both control levels were reanalyzed on the CS-2500 in the afternoon on study days 1 and 3. Thus, internal quality control was performed on six occasions on the CS-2500 and on four occasions on the NOVAE II during the study period. For the CS-2500, PT quality control was performed using CONTROL N (lot 507961; Siemens Healthineers) as the normal control and CONTROL P (lot 556753; Siemens Healthineers) as the pathological control. aPTT quality control was performed using CONTROL N (lot 507964; Siemens Healthineers) as the normal control and Dade Ci-Trol 2 (lot 548554; Siemens Healthineers) as the pathological control. NOVAE II quality control was performed using MTI-1320 N.Control (lot 31166440) and MTI-1321 A.Control (lot 22009709) as the normal and pathological control materials, respectively. Control results were evaluated against the manufacturer-assigned, lot- and instrument-specific acceptance ranges, and all QC results preceding study measurements were within the predefined acceptable limits. The CS-2500 participated in the KBUDEK 2026 Coagulation External Quality Assessment Program (Period 7), with external quality-assessment samples analyzed monthly. During the March 2026 distribution corresponding to the study period, the performance of PT, INR, and aPTT was rated as satisfactory by the program provider. The NOVAE II was not enrolled in an external quality-assessment program because it was installed temporarily for the 4-day evaluation period.

### 2.3. Reference Intervals and Categorical Classification

The reference intervals used for the CS-2500 in patients aged 18–150 years were PT 10–14 s, INR 0.8–1.2, and aPTT 22.1–30.2 s, based on established laboratory guidelines and long-standing routine laboratory practice. For the NOVAE II, the ranges provided in the reagent package inserts were used (PT 11.6–16.4 s, INR 0.8–1.2, and aPTT 22–38 s). These intervals were used only for exploratory normal/abnormal classification and were not treated as diagnostic gold standards. Because PT and aPTT thresholds differed between systems, categorical discordance reflects both measurement differences and differences in classification thresholds.

### 2.4. Statistical Analysis

All statistical analyses were performed using MedCalc Statistical Software version 23.2.6 (MedCalc Software Ltd., Ostend, Belgium; https://www.medcalc.org; 2025). Valid paired measurements were analyzed separately for PT, INR, and aPTT. Continuous variables were summarized using mean, standard deviation, median, quartiles, minimum, and maximum values. Method comparison was conducted according to CLSI EP09c recommendations for measurement procedure comparison and bias estimation using patient samples [13]. Pearson correlation was used as the primary measure of linear association. Spearman’s rank correlation was retained as a secondary robustness analysis because observations were concentrated near the routine reference range with a small number of higher values; neither correlation coefficient was interpreted as evidence of agreement.

Passing–Bablok regression was performed with CS-2500 values on the *x*-axis and NOVAE II values on the *y*-axis, without transformation. The intercept and slope were reported with 95% confidence intervals (CIs). Constant bias was considered statistically supported only when the 95% CI for the intercept excluded 0, and proportional bias only when the 95% CI for the slope excluded 1.

Bland–Altman analysis used the paired difference (NOVAE II minus CS-2500) plotted against the mean of the two measurements. Mean difference and 95% limits of agreement (LoA) were calculated as mean difference ± 1.96 SD of the paired differences; 95% CIs were also calculated for the mean difference and approximate 95% CIs for the lower and upper LoA. Mean relative difference (%) was calculated as the mean of 100 × (NOVAE II − CS-2500)/CS-2500 across paired samples. No clinical equivalence margin or allowable bias limit was prespecified; Bland–Altman findings were therefore interpreted as analytical differences rather than evidence of clinical interchangeability. Categorical agreement was assessed using analyzer-specific reference intervals and summarized by observed agreement and Cohen’s kappa with 95% nonparametric bootstrap CIs based on 10,000 resamples; this analysis was considered exploratory because abnormal findings were infrequent and PT/aPTT classification thresholds differed between systems.

## 3. Results

After verification of the dataset, analyses were performed using all valid paired measurements available for each parameter. PT and INR analyses included 50 paired measurements, while aPTT analysis included 54 paired measurements after exclusion of invalid or missing aPTT results.

### 3.1. Descriptive Findings

Descriptive statistics for PT, INR, and aPTT measurements are presented in Table 1. For PT, across 50 paired measurements, the CS-2500 yielded a mean value of 12.05 ± 2.18 s (median 11.80; range 10.40–25.50 s), whereas the Tokra NOVAE II produced a mean value of 13.32 ± 2.19 s (median 13.20; range 10.30–25.40 s). These findings indicate that the Tokra NOVAE II systematically generated higher PT values relative to the CS-2500, a pattern consistent with the +1.27 s mean difference reported in the study abstract.

For INR, the discrepancy between the two analyzers was comparatively modest. The CS-2500 produced a mean INR of 1.02 ± 0.20 (median 1.00; range 0.88–2.23), whereas the NOVAE II yielded a mean of 1.05 ± 0.22 (median 1.01; range 0.78–2.28).

For aPTT, 54 paired measurements were evaluated. The CS-2500 demonstrated a mean value of 25.63 ± 3.16 s (median 25.00; range 20.90–37.60 s), whereas the NOVAE II showed a mean of 27.94 ± 3.80 s (median 27.20; range 22.70–42.00 s). The wider dispersion observed for aPTT was evaluated further using Passing–Bablok regression and Bland–Altman analysis.

Overall, NOVAE II produced higher mean values than the CS-2500 for all three parameters. Using analyzer-specific reference intervals, abnormal findings were uncommon: CS-2500 PT 2/50, INR 2/50, and aPTT 7/54; NOVAE II PT 8/50, INR 4/50, and aPTT 1/54. The maximum observed INR was 2.28 and the maximum aPTT was 42.0 s, indicating limited representation of markedly pathological or therapeutic measurement ranges.

### 3.2. Method-Comparison Analyses

Method-comparison statistics are summarized in Table 2.

For PT, Passing–Bablok regression yielded an intercept of −1.301 (95% CI: −5.727 to 1.982) and a slope of 1.224 (95% CI: 0.944 to 1.600). Because the intercept CI included 0 and the slope CI included 1, neither constant nor proportional bias was statistically supported by the regression analysis. Pearson r was 0.945 and Spearman rho was 0.770, indicating a strong association but not establishing agreement. Bland–Altman analysis showed a mean difference of +1.27 s (95% CI: +1.07 to +1.48), with a lower 95% LoA of −0.15 s (approximate 95% CI: −0.50 to +0.21) and an upper 95% LoA of +2.69 s (approximate 95% CI: +2.34 to +3.05). The mean relative difference was 10.78%.

For INR, the Passing–Bablok intercept was −0.165 (95% CI: −0.480 to 0.136) and the slope was 1.200 (95% CI: 0.889 to 1.500). Both confidence intervals included the respective null values, so constant or proportional bias was not statistically supported by the regression analysis. Pearson r was 0.951 and Spearman rho was 0.727. Bland–Altman analysis showed a mean difference of +0.023 (95% CI: +0.003 to +0.043), with a lower 95% LoA of −0.112 (approximate 95% CI: −0.146 to −0.078) and an upper 95% LoA of +0.159 (approximate 95% CI: +0.125 to +0.193). The mean relative difference was 2.16%.

For aPTT, the Passing–Bablok intercept was 2.792 (95% CI: −2.816 to 9.809) and the slope was 0.982 (95% CI: 0.687 to 1.208). The confidence intervals included 0 and 1, respectively, and therefore did not provide statistically supported evidence of constant or proportional bias. Pearson r was 0.843 and Spearman rho was 0.776. Bland–Altman analysis showed a mean difference of +2.32 s (95% CI: +1.76 to +2.88), with a lower 95% LoA of −1.70 s (approximate 95% CI: −2.66 to −0.74) and an upper 95% LoA of +6.33 s (approximate 95% CI: +5.37 to +7.29). The mean relative difference was 9.13%. Taken together, Passing–Bablok regression did not demonstrate statistically supported constant or proportional bias for any parameter, whereas Bland–Altman analysis identified positive average paired differences and assay-specific widths of the LoA. Because clinical acceptability limits were not prespecified, these findings characterize analytical comparability within the observed range and should not be interpreted as evidence of clinical interchangeability.

**Figure 1:**
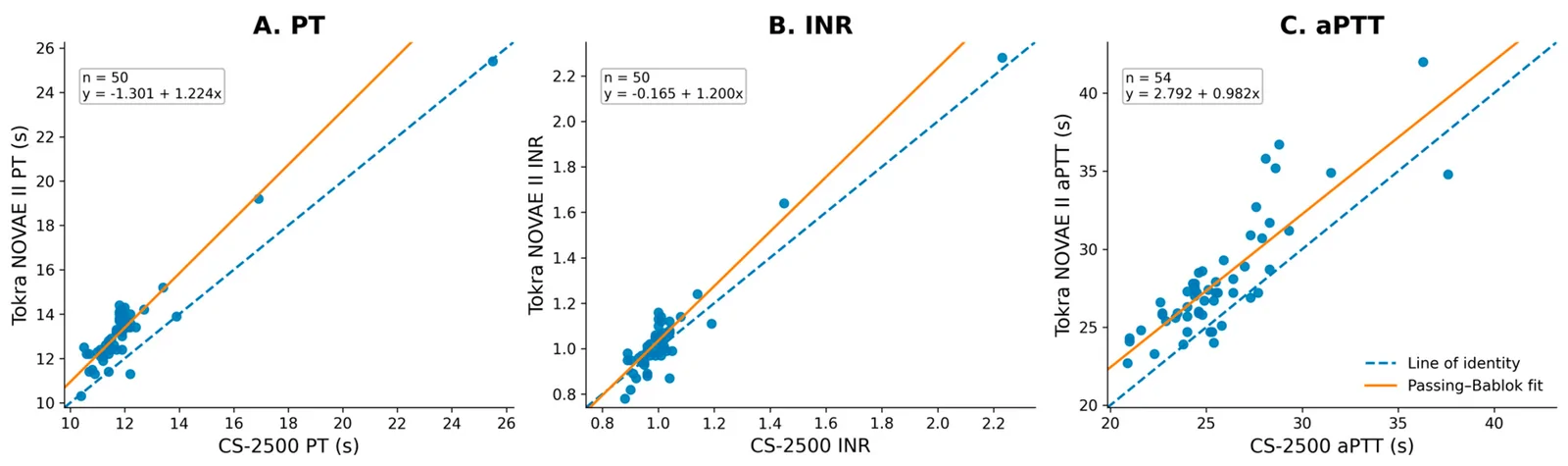
Passing–Bablok method-comparison plots for (**A**) PT, (**B**) INR, and (**C**) aPTT. CS-2500 values are plotted on the *x*-axis as the comparator measurements and NOVAE II values on the *y*-axis. The dashed line represents the line of identity, and the solid line represents the Passing–Bablok regression line.

Regression equation: y = intercept + slope × x, where x represents CS-2500 values and y represents NOVAE II values. Paired differences were calculated as NOVAE II minus CS-2500; 95% LoA = mean difference ± 1.96 SD. Values in parentheses beside each LoA are approximate 95% CIs for the estimated lower or upper limit. Mean relative difference = mean [100 × (NOVAE II − CS-2500)/CS-2500]. CI, confidence interval; LoA, limits of agreement. PT and aPTT are expressed in seconds (s). The Passing–Bablok method-comparison plots for PT, INR, and aPTT are shown in Figure 1A–C.

For PT, most observations were clustered within approximately 10–16 s, with only a few higher values. The fitted regression line deviated visually from the line of identity at the upper end of the observed range; however, because the intercept CI included 0 and the slope CI included 1, the plot should not be interpreted as statistically supported constant or proportional bias.

For INR, most observations were concentrated within the 0.8–1.2 range, with only a small number of higher values. The regression line remained relatively close to the line of identity across the densely sampled portion of the observed range, consistent with the small mean paired difference; however, the limited number of elevated INR values restricts interpretation beyond the routine range.

For aPTT, the data showed greater scatter than for PT or INR, particularly within approximately 25–35 s. Although the fitted line appeared vertically displaced from the line of identity, the wide intercept CI included 0 and the slope CI included 1; therefore, the visual pattern was interpreted as imprecise analytical dispersion rather than evidence of statistically supported constant or proportional bias.

Overall, the Passing–Bablok plots illustrate that the measurement range was dominated by values close to routine reference intervals, with comparatively few elevated observations. This limited range should be considered when interpreting the regression estimates and their confidence intervals.

**Figure 2:**
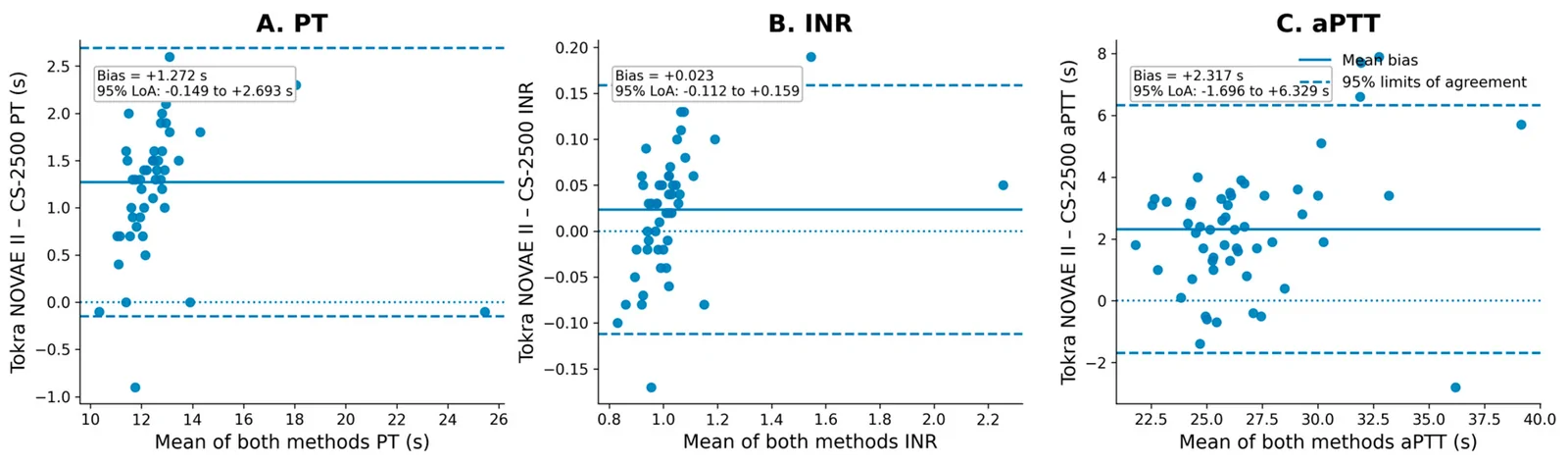
Bland–Altman plots for (**A**) PT, (**B**) INR, and (**C**) aPTT. Differences were calculated as NOVAE II minus CS-2500. The solid horizontal line represents the mean paired difference, and the dashed lines represent the 95% limits of agreement.

The Bland–Altman plots are presented in Figure 2A–C and illustrate the direction and dispersion of paired differences across the observed measurement range.

For PT, the mean paired difference was +1.272 s, with 95% LoA from −0.149 to +2.693 s. Most paired differences were positive, indicating that the NOVAE II tended to yield higher PT values within the observed range; however, clinical acceptability of this magnitude of difference was not prespecified.

For INR, the mean paired difference was +0.023, with 95% LoA from −0.112 to +0.159. The differences were closely distributed around the mean difference within the predominantly near-normal range, but the limited number of elevated INR values restricts conclusions at therapeutic decision points.

For aPTT, the mean paired difference was +2.317 s, with the widest 95% LoA among the three parameters (−1.696 to +6.329 s). The wider dispersion may be relevant near clinical decision thresholds, but the present dataset does not contain sufficient therapeutic-range or anti-Xa-linked samples to determine whether these differences would alter heparin dose adjustment.

Taken together, Bland–Altman analysis showed the smallest mean difference and narrowest LoA for INR and the widest LoA for aPTT. These observations describe analytical differences within the sampled range and do not establish clinical interchangeability.

### 3.3. Categorical Agreement

Categorical agreement results are summarized in Table 3. For PT, 42 of 50 samples were classified as normal and 2 as abnormal by both analyzers; 6 were normal on CS-2500 but abnormal on NOVAE II, and none showed the reverse pattern. Observed agreement was 88.0%, with Cohen’s kappa 0.359 (95% bootstrap CI: 0.000–0.730). The broad CI and low prevalence of abnormal findings warrant cautious interpretation.

For INR, 46 of 50 samples were concordantly classified as normal and 2 as abnormal, while 2 were normal on CS-2500 but abnormal on NOVAE II. Observed agreement was 96.0%, with Cohen’s kappa 0.648 (95% bootstrap CI: 0.000–1.000). The wide CI reflects the small number of abnormal classifications despite the high raw agreement.

For aPTT, 47 of 54 samples were concordantly classified as normal and 1 was abnormal. Six samples were abnormal on CS-2500 but normal on NOVAE II, and none showed the reverse pattern. Observed agreement was 88.9%, with Cohen’s kappa 0.225 (95% bootstrap CI: 0.000–0.640). Because the upper reference limits differed substantially between systems (30.2 s versus 38 s), this discordance reflects both analytical differences and different classification thresholds.

The categorical analysis was exploratory. Kappa estimates were imprecise because abnormal results were uncommon, and analyzer-specific PT/aPTT reference intervals used different thresholds. Accordingly, categorical concordance should not be interpreted as evidence for or against clinical interchangeability.

## 4. Discussion

The present study compared PT, INR, and aPTT results generated by the CS-2500 and NOVAE II analyzer–reagent systems using paired routine plasma specimens. Passing–Bablok confidence intervals did not demonstrate statistically supported constant or proportional bias for any of the three parameters, whereas Bland–Altman analysis showed positive average paired differences and assay-specific widths of the limits of agreement. Because no clinical acceptability margins were prespecified, these findings should be interpreted as analytical comparability data within the observed measurement range rather than evidence of clinical interchangeability.

INR showed the smallest mean paired difference and narrowest LoA, whereas aPTT showed the widest LoA. The INR standardization framework, which incorporates the international sensitivity index (ISI) and mean normal prothrombin time, may contribute to reduced cross-system variability; however, the present design cannot establish ISI correction as the cause of the observed pattern because analyzer, reagent formulation, and calibration differed simultaneously. In contrast, aPTT lacks a comparable universal standardization framework and is well recognized to be sensitive to reagent composition, including the contact activator and phospholipid system [14,15]. These factors provide plausible context for the observed differences but should not be interpreted as causal findings from this study.

The direction and magnitude of the paired differences are broadly consistent with previous coagulation analyzer comparison studies, which frequently report positive correlations alongside assay-specific biases and wider dispersion for aPTT than for INR [6,7,8,9,10]. The contribution of the present study is therefore not a novel general hierarchy of coagulation assays, but an independent paired patient-sample evaluation of the locally implemented CS-2500 and NOVAE II analyzer–reagent systems, including regression uncertainty, Bland–Altman differences, and threshold-dependent categorical discordance.

The results also reinforce the distinction between association and agreement. Pearson and Spearman coefficients describe the strength of association but do not establish numerical equivalence between systems. Likewise, categorical agreement depended not only on measured values but also on analyzer-specific reference intervals. This was particularly evident for aPTT, for which the upper reference limits differed substantially between systems. The broad bootstrap CIs for kappa further reflect the low prevalence of abnormal findings and underscore the need for cautious interpretation of chance-corrected categorical statistics in this dataset [12,16,17].

The clinical implications should therefore be interpreted conservatively. The aPTT mean paired difference of approximately +2.32 s and LoA of approximately −1.70 to +6.33 s could potentially affect interpretation near a clinical decision threshold. However, the study did not include sufficient therapeutic-range aPTT values, anti-Xa-linked samples, or anticoagulant-treatment information to determine whether heparin dose adjustment would differ between systems. Similarly, the Passing–Bablok equations should be regarded as descriptive method-comparison outputs and should not be used as clinical conversion equations because the abnormal range was sparsely sampled and no independent validation cohort was available. The current data also do not support derivation of a universal aPTT percentage or ratio for harmonization across analyzers.

From a laboratory quality-management perspective, the findings support local patient-sample verification whenever multiple coagulation analyzer–reagent systems are used in parallel [3]. Analyzer-specific reference intervals, parallel quality-control procedures, and, when aPTT is used for unfractionated heparin monitoring, locally established analyzer/reagent-specific therapeutic ranges remain preferable to assuming numerical equivalence across systems. These recommendations reflect the limitations of cross-platform standardization and should not be interpreted as evidence that either analyzer is clinically superior.

This study has several limitations. First, it was a single-center analysis with a modest sample size (50 paired PT/INR measurements and 54 paired aPTT measurements). More importantly, most observations were within or close to routine reference ranges: the maximum INR was 2.28 and the maximum aPTT was 42.0 s, and markedly pathological or therapeutic-range values were uncommon. Second, clinical diagnosis, coagulation-disorder status, and anticoagulant exposure were not retained in the analytical dataset, preventing clinically relevant subgroup analyses. Third, no clinical equivalence margin or allowable bias threshold was prespecified, so the Bland–Altman findings cannot establish clinical interchangeability. Fourth, the exploratory categorical analysis used analyzer-specific reference intervals, and the differing PT/aPTT thresholds confounded measurement differences with classification differences. Fifth, the analyzer–reagent combinations differed in multiple components simultaneously, preventing attribution of the observed differences to a single analytical factor. Future multicenter studies should intentionally enrich the sample set with therapeutic anticoagulation, factor deficiencies, liver disease, lupus anticoagulant, and other clinically important abnormal ranges and should incorporate independently defined clinical decision criteria.

In conclusion, the CS-2500 and NOVAE II analyzer–reagent systems showed positive analytical associations for PT, INR, and aPTT, but the magnitude and dispersion of paired differences varied by assay. Passing–Bablok regression did not provide statistically supported evidence of constant or proportional bias within the observed range, while Bland–Altman analysis showed the narrowest dispersion for INR and the widest for aPTT. Given the limited representation of pathological and therapeutic values and the absence of prespecified clinical equivalence criteria, the present findings support local analytical verification and analyzer-specific interpretation rather than an assumption of clinical interchangeability.

## Figures and Tables

**Table 1:** Descriptive statistics of paired PT, INR, and aPTT measurements.

Parameter	*n*	CS-2500 Mean ± SD	CS-2500 Median (Min–Max)	Tokra NOVAE II Mean ± SD	Tokra NOVAE II Median (Min–Max)
**PT**	50	12.05 ± 2.18	11.80 (10.40–25.50)	13.32 ± 2.19	13.20 (10.30–25.40)
**INR**	50	1.02 ± 0.20	1.00 (0.88–2.23)	1.05 ± 0.22	1.01 (0.78–2.28)
**aPTT**	54	25.63 ± 3.16	25.00 (20.90–37.60)	27.94 ± 3.80	27.20 (22.70–42.00)

CS-2500 refers to the Sysmex CS-2500 System using Siemens reagents. PT and aPTT results are expressed in seconds (s).

**Table 2:** (**A**) Passing–Bablok regression and association statistics for NOVAE II versus the CS-2500 comparator system. (**B**) Bland–Altman statistics for NOVAE II minus CS-2500, including 95% confidence intervals for the mean difference and both limits of agreement.

(A)					
Parameter	*n*	Passing–Bablok Intercept (95% CI)	Passing–Bablok Slope (95% CI)	Pearson r	Spearman rho
**PT**	50	−1.301 (−5.727 to 1.982)	1.224 (0.944 to 1.600)	0.945	0.770
**INR**	50	−0.165 (−0.480 to 0.136)	1.200 (0.889 to 1.500)	0.951	0.727
**aPTT**	54	2.792 (−2.816 to 9.809)	0.982 (0.687 to 1.208)	0.843	0.776
**(B)**					
**Parameter**	***n***	**Mean Difference (95% CI)**	**Lower 95% LoA (95% CI)**	**Upper 95% LoA (95% CI)**	**Mean relative difference (%)**
**PT**	50	+1.27 s (+1.07 to +1.48)	−0.15 s (−0.50 to +0.21)	+2.69 s (+2.34 to +3.05)	10.78%
**INR**	50	+0.023 (+0.003 to +0.043)	−0.112 (−0.146 to −0.078)	+0.159 (+0.125 to +0.193)	2.16%
**aPTT**	54	+2.32 s (+1.76 to +2.88)	−1.70 s (−2.66 to −0.74)	+6.33 s (+5.37 to +7.29)	9.13%

**Table 3:** Exploratory categorical agreement according to analyzer-specific reference intervals, with 95% bootstrap confidence intervals for Cohen’s kappa.

Parameter	*n*	Both Normal	Both Abnormal	CS-2500 Normal/Tokra Abnormal	CS-2500 Abnormal/Tokra Normal	Observed Agreement	Cohen’s Kappa (95% Bootstrap CI)
**PT**	50	42	2	6	0	88.0%	0.359 (0.000–0.730)
**INR**	50	46	2	2	0	96.0%	0.648 (0.000–1.000)
**aPTT**	54	47	1	0	6	88.9%	0.225 (0.000–0.640)

CS-2500 reference intervals: PT 10–14 s, INR 0.8–1.2, aPTT 22.1–30.2 s. NOVAE II reference intervals: PT 11.6–16.4 s, INR 0.8–1.2, aPTT 22–38 s. Because PT and aPTT thresholds differed between systems, kappa reflects both measurement differences and threshold-dependent classification differences.

## Data Availability

The original contributions presented in this study are included in the article. Further inquiries can be directed to the corresponding author.
